# At the core of global bioenergy: polyamines and transglutaminases in chloroplasts

**DOI:** 10.1007/s00726-026-03518-0

**Published:** 2026-03-24

**Authors:** Donatella Serafini-Fracassini, Stefano Del Duca

**Affiliations:** 1 Department of Biological, Geological and Environmental Sciences, University of Bologna, 40126 Bologna, Italy; 2 Interdepartmental Centre for Agri-Food Industrial Research, University of Bologna, 40126 Bologna, Italy

**Keywords:** Chloroplast, Photosynthesis, Polyamines, Stress responses, Thylakoids, Transglutaminases

## Abstract

Chloroplasts are the energy factories of photosynthetic life. The energy and the plant biomass of the planet depend on the activity of chloroplasts. Their efficiency results from a delicate balance between membrane organization, metabolic regulation and the maintenance of protein homeostasis. Among the molecular factors that support chloroplast structure and function, polyamines (PAs) and transglutaminases (TGases) have emerged as key regulators of photosynthetic performance and stress tolerance. PAs stabilize thylakoid membranes, influence proton-motive force partitioning, modulate chlorophyll biosynthesis and protect the photosynthetic apparatus from oxidative damage, thereby contributing to increased photoprotection and delayed senescence. TGases, particularly the plastid-localized isoforms, catalyze the covalent conjugation of PAs to stromal and thylakoid proteins, including RuBisCo and light-harvesting complexes (LHC). These reactions affect protein stability, supramolecular assembly, and the dynamic remodeling of LHC. Light-, pH- and redox-dependent activation of TGases links PA dependent protein modification to the regulation of photosynthetic efficiency and stress responses. This review integrates biochemical, structural and physiological evidence to highlight how PAs and TGases operate at the interface of light-to-chemical energy conversion. Both are involved in membrane organisation and maintaining protein quality. Understanding this molecular network provides new perspectives for improving plant performance, enhancing tolerance to abiotic stress and sustaining biomass production, notably that of agriculture interest, under changing environmental conditions.

## Introduction

It is remarkable that a single plant organelle, the chloroplast, is responsible for most of the solar energy conversion on Earth, both in ancient and present geological eras. Solar radiation represents the primary direct energy source sustaining the biosphere. Most of the energy exploited today, whether derived from fossil fuels or modern renewable systems, can be traced back to biomass originated from the sun. Solar energy is also utilised directly for food production, whether plant- or animal-based. Cultivated plants can be consumed as such or transformed and they are essential for the survival of global population. In the nature, the conversion of solar energy into stable chemical energy occurs inside chloroplasts through a highly complex metabolic system which remains extremely difficult to reproduce artificially (Dukes et al. 2003). This review integrates current evidence on polyamine (PA) metabolism and chloroplast-localised functions and discusses emerging mechanistic frameworks linking PAs and transglutaminases (TGases) in the context of chloroplast development, photoprotection, and stress resilience.

## Chloroplasts

**Fig. 1:**
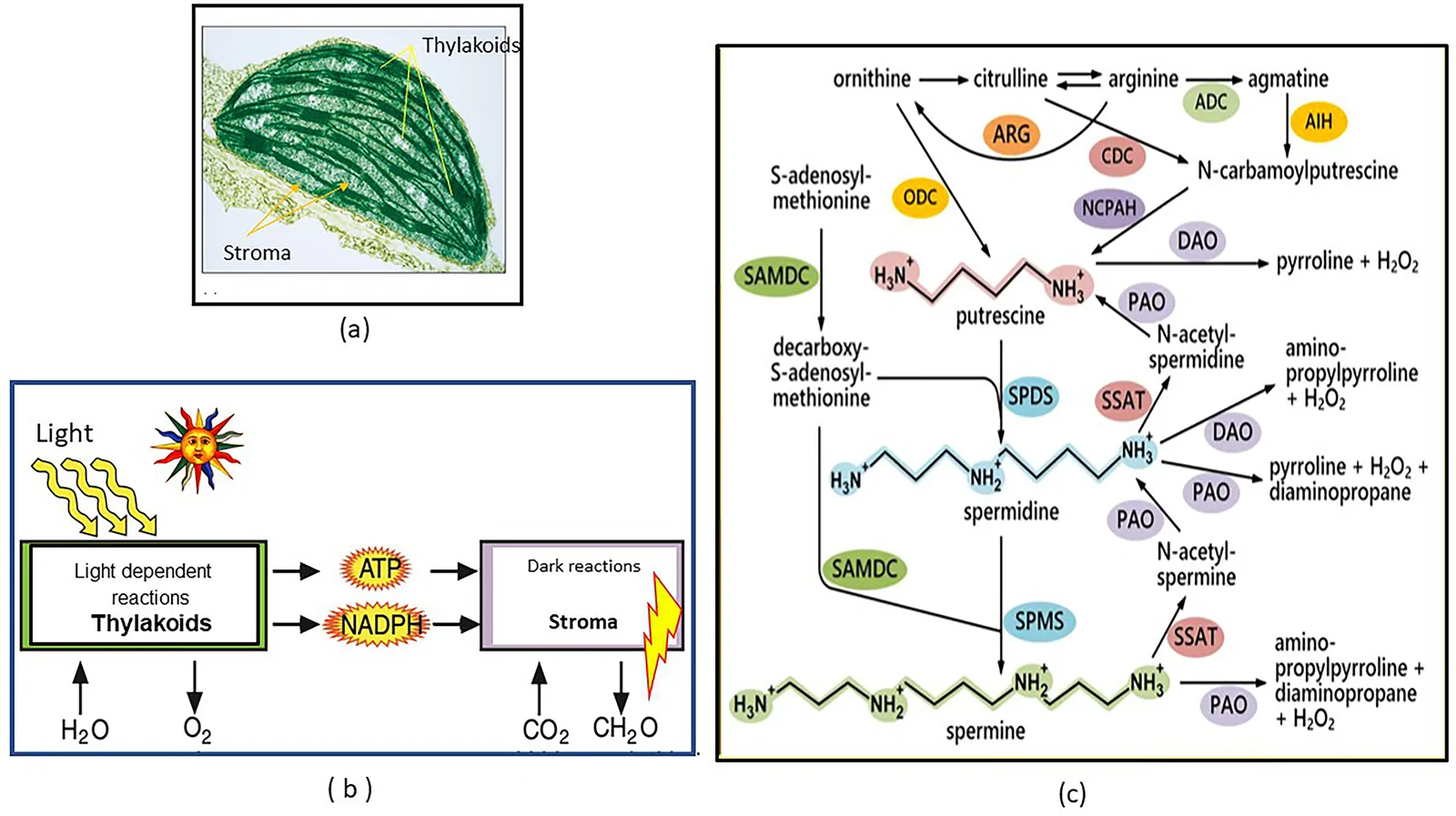
**a** The chloroplast, enclosed in a double membrane, is composed by a soluble fraction, the stroma, and by a membrane fraction, the thylakoids, which can be as single membrane and stalked in piles of membranes, the grana (more in detail in Fig. 2b). Thylakoids have intramembrane light harvesting photosystems (LHC I and II) to which chlorophyll and other pigments are linked. This causes the green color of the chloroplast. Thylakoids are immersed in the soluble fraction, the stroma containing the enzyme RuBisCo (ribulose-1,5-bisphosphate carboxylase/oxygenase) responsible of the fixation of CO_2_ into carbohydrates, independently from the light presence. This is the most abundant enzyme in the heart. **b** Light is requested for the synthesis of ATP and NAPDH in the thylakoid membranes (Light-dependent reactions) utilizing H_**2**_O and releasing O_**2**_. ATP and NADPH are thereafter used by stroma enzyme RuBisCo in order to fix CO_**2**_ into carbohydrates (dark reactions). ATP-ADP and NADP^**+**^-NADPH diffuse between the two zones. Thus, light-dependent photosynthetic reactions generate chemical energy, which is transferred through thylakoid membranes in the form of ATP and NADPH. These energy-rich molecules are then used in the stroma to synthesize carbohydrates. In this way, light energy is converted into stable chemical energy that supports the metabolism of photosynthetic organisms. Plant biomass so produced in the course of geological eras became fossilized giving rise to gas or oil utilized by humans to recover part of the energy there stored. The actual plants also contain energy and most of it is stored in seeds. **c** Anabolic and catabolic pathways of PAs in plants and their direct precursors: ornithine, and arginine. PAs biosynthesis: the production of Put can be catalyzed by ADC indirectly form arginine, by ODC directly from ornithine, or indirectly by CDC from citrulline. In the next step, SPDS is responsible for biosynthesis of Spd from Put, and SPMS participates in the production of Spm from Spd. During this process, decarboxylated S-adenosylmethionine (dcSAM) serves as the aminopropyl donor for the synthesis of Spd and Spm. The enzyme S-adenosylmethionine decarboxylase (SAMDC) catalyzes the conversion of S-adenosylmethionine into its decarboxylated form, thereby providing the required precursor for higher PA biosynthesis. In PA catabolism and back-conversion, diamine oxidase (DAO) catalyzes the oxidation of Put, generating Δ^1^-pyrroline and H₂O₂, as well as other metabolites. Polyamine oxidase (PAO) participates in Spd and Spm oxidation, producing H₂O₂, 1,3-diaminopropane, and either 1-pyrroline or 1-(3-aminopropyl)-pyrroline. PAO also participates in the back-conversion of PAs, whereby Spm is converted to Spd, and subsequently to Put. Abbreviations: *ADC* arginine decarboxylase, *CDC* citrulline decarboxylase, *DAO* diamine oxidase, *Dsam* decarboxylated s-adenosylmethionine, *ODC* ornithine decarboxylase, *PAO* polyamine oxidase, *Put* putrescine, *Sam* s-adenosylmethionine, *SAMDC* s-adenosylmethionine decarboxylase, *Spd* spermidine, *SPDS* spermidine synthase, *Spm* spermine, *SPMS* spermine synthase

Among other plastids (Altamura et al 2024), chloroplasts are organelles present in all photosynthetic cells (Fig. 1a); they are metabolically complex and their biogenesis is fundamental for determining the photosynthetic capacity of plants. They represent one of the major plastid types; in terrestrial plants, chloroplasts are typically lens-shaped (lenticular) organelles. A commonly used, internally consistent size estimate is 5–10 µm along the major (long) axis and ~ 2–3 µm across the minor (short) axis. Each chloroplast contains a circular DNA molecule and is enclosed by a double membrane that surrounds an unstructured stroma. Within the stroma, the key enzyme ribulose-1,5-bisphosphate carboxylase/oxygenase (RuBisCo) catalyses CO₂ fixation, generating carbohydrate precursors even in the absence of light (Fig. 1b). The stroma also hosts the biosynthetic and regulatory components of the photosynthetic machinery.

**Fig. 2:**
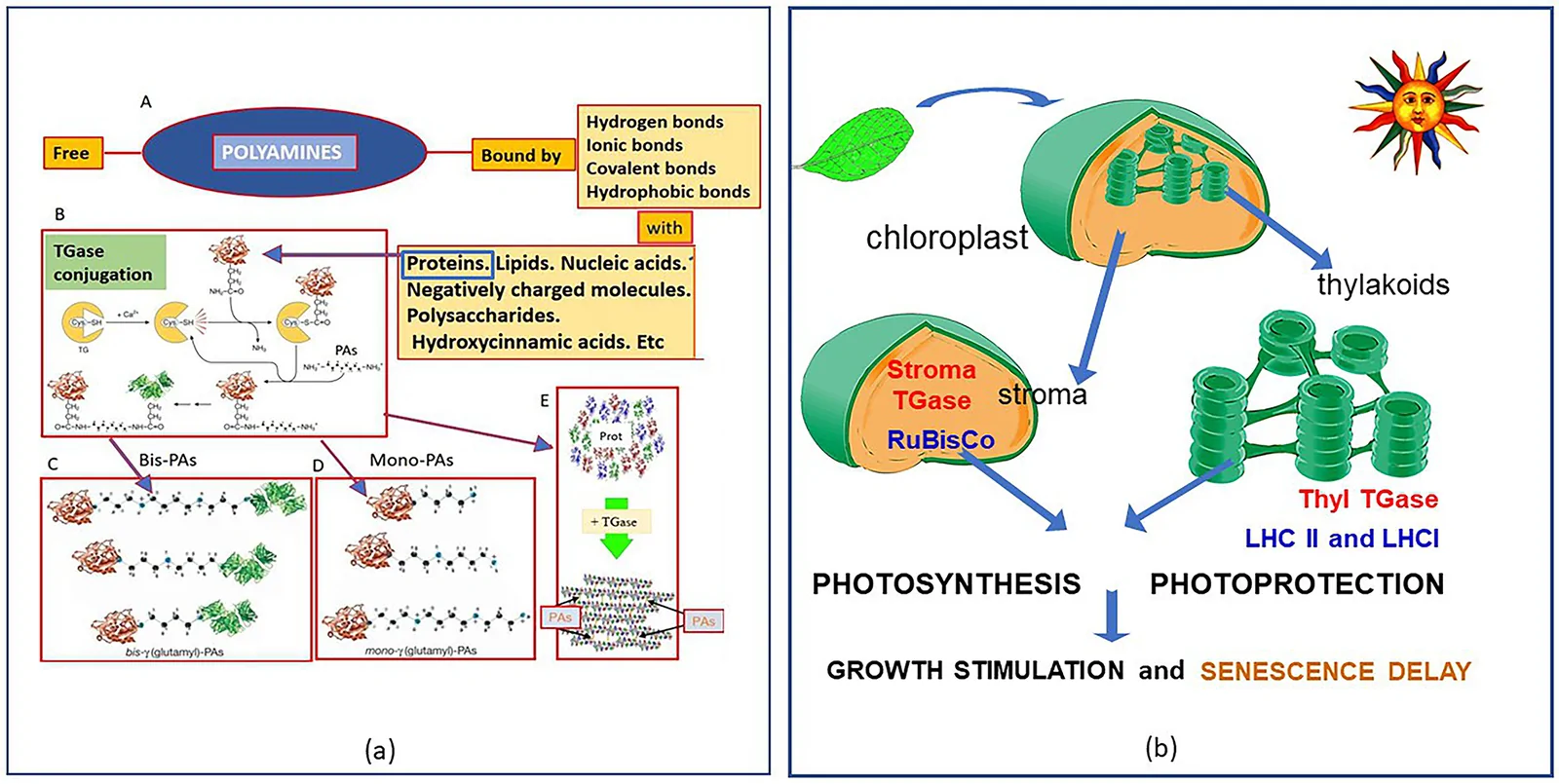
**a** Polyamines (PAs) may occur in a free form or bound to other molecules through different types of interactions, including hydrogen bonds, ionic or hydrophobic interactions, and covalent linkages. As illustrated in the schematic box (A), PAs can associate with a wide range of molecular partners. Proteins, in particular, can serve as substrates for transglutaminase (TGase). (B) Through a Ca^2^⁺-dependent transamidation reaction, TGase links PAs to glutamyl residues on target proteins, generating mono-γ-glutamyl-PAs (D) and, after a second transamidation step, bis-γ-glutamyl-PAs (C). When multiple TGase reactions occur across different proteins, a network of PA-bridged protein aggregates is formed (E). **b** In the chloroplast the enzyme transglutaminase (TGase) is present under two main isoenzyme forms located either in stroma or in thylakoids, having respectively as protein substrates RuBisCo and the photosynthetic complexes (light harvesting complexes, i.e. LHCI and LHCII). The catalysis of the binding of PAs to these protein substrates in the presence of light, favour photosynthesis and photoprotection, causing an increase of growth and senescence delay of the plant

Photosynthetic reactions are primarily localised in the thylakoid system, organised into a continuous membrane network composed of stacked grana thylakoids and unstacked stroma lamellae (Fig. 1a and 2b). In the presence of light, chlorophyll molecules embedded in the thylakoids, capture photons and initiate the conversion of light energy into chemical energy in the form of ATP and reducing power in the form of NADPH (Fig. 1b). These energy carriers fuel the Calvin–Benson cycle, enabling RuBisCo to assimilate CO₂ and supporting multiple interconnected metabolic pathways. (Jardine et al. 2025).

A specific plastid form, the etioplast, characterises plants grown in darkness. Etioplasts contain large prolamellar bodies, membrane aggregates with a semi-crystalline tubular net (prothylakoids) that host the chlorophyll precursor protochlorophyllide. Upon light exposure, etioplasts differentiate into chloroplasts through the synthesis of chlorophyll bound to newly formed thylakoid membranes. During photomorphogenesis, prolamellar bodies reorganise into stroma and grana thylakoids to form chloroplasts (Biswal et al. 2003). Immature proplastids undergo similar developmental transitions.

Chloroplasts are the central hubs of solar energy conversion in plants and therefore sustain most of Earth’s biomass, food chains, and energy foundations. Their activity dominates the global utilisation of solar energy, a resource that ultimately underpins both natural ecosystems and the majority of energy sources exploited by human societies, including fossil fuels and modern renewable technologies (Burrington 2024; Mensah and Yankson 2025). The evolutionary origin of chloroplasts traces back to the integration of a cyanobacterial endosymbiont into a eukaryotic host, an event that enabled the emergence of oxygenic photosynthesis (Fig. 1b) and reshaped atmospheric composition, due to oxygen availability, and global biogeochemical cycles (Zimorski et al. 2014; Sato 2011). This biological innovation led to the diversification of photosynthetic eukaryotes and the formation of what is now recognised as the plant kingdom. The capture of light energy and its conversion into chemical energy, under form of carbohydrates, accompanied by the release of molecular oxygen from water and of utilisation of carbon dioxide (Fig. 1b), started about 2.8 billion years ago, and chloroplasts profoundly altered Earth’s atmosphere, enabled aerobic metabolism, and supported the evolution of complex organisms, including humans. Because they operate at the interface between environmental energy input and cellular metabolism, chloroplasts are highly sensitive to fluctuations in light intensity, redox state, temperature, and water availability. Maintaining their structural and functional integrity under such conditions is essential for plant productivity, particularly under climate change and increasing global food demand. Modern agriculture faces the challenge of enhancing plant growth and yield while mitigating stress factors such as drought and soil salinity, which are affecting vast agricultural regions around the world. As a result, research efforts increasingly focus on photosynthesis and its regulatory mechanisms, as photosynthetic optimisation represents a promising strategy to improve biomass production and crop performance especially under suboptimal conditions.

Recent advances in chloroplast proteostasis, signalling, and photoprotection highlight the organelle’s dynamic regulatory capacity (Sun and Jarvis 2023; Rochaix 2022). Among the molecular regulators involved, PAs have emerged as key modulators of chloroplast development, thylakoid organisation, photosynthetic efficiency, and stress tolerance.

## Polyamines and their role in plastids

The major plant PAs, putrescine (Put), spermidine (Spd), spermine (Spm), and thermospermine (tSpm). t-Spm is conserved across land plants and involved in development, but current evidence does not support its specific accumulation or function within chloroplasts. Further studies and investigations would be necessary to clarify its presence and role in chloroplasts. In plant cells, PAs occur in free, bound (to nucleic acids and lipids) and conjugated (to protein) forms and share several physiological effects with PAs of animal origin. They participate in gene regulation, ion homeostasis, membrane stabilisation, and ROS responses (Tiburcio et al. 2014; Podlešáková et al. 2019; Alcázar et al. 2020). Their role in plant development was first observed in *Helianthus tuberosus* cultured in vitro (Bertossi et al. 1965) and later confirmed in many plant species, where they are also involved in pollen-pistil interaction during fertilization (Gentile et al 2012). Unlike animals, however, plants contain plastids, and PAs have specific functions within these organelles, whose metabolic complexity is unparalleled due to their ability to convert light energy into chemical energy. Evidence for PA involvement in the formation and function of the photosynthetic apparatus was first provided by the detection of PAs in isolated chloroplasts of *Euglena gracilis* (Bagni and Serafini-Fracassini 1974) and by the discovery of enzymes involved in PA biosynthesis (Torrigiani et al. 1987; Borrell et al. 1995; Bortolotti et al. 2004) and oxidation (Andreadakis and Kotzabasis 1996). Their presence in plastids has long been documented, with roles reported in chloroplast differentiation, thylakoid architecture, and protection from photoinhibition (Bagni and Serafini-Fracassini 1974; Beigbeder et al. 1995; Sobieszczuk-Nowicka et al. 2007a). Although decades of research indicate that PAs contribute to chloroplast stability and function, the underlying molecular mechanisms remain only partially resolved, with hypotheses ranging from photosynthetic complex stabilisation to modulation of proton motive force, chlorophyll biosynthesis and redox buffering. The plants PA biosynthetic pathway has been extensively characterised and includes steps that are not present in animals (Fig. 1c) (Bagni & Tassoni 2001). Putrescine can be synthesised via ornithine decarboxylase (ODC), via arginine decarboxylase (ADC) through a two-step pathway, or via citrulline (CDC) (Smith et al. 1979; Mengoli et al. 1987). *Arabidopsis thaliana* possesses two ADC genes, *AtADC1* and *AtADC2*, and the double mutant is lethal (Urano et al. 2005). Enhanced Put biosynthesis can improve plant growth and salinity tolerance, as demonstrated for AtADC2-dependent pathways (Jasso-Robles et al. 2024). The ADC pathway is typical of plants and some fungi and is strongly up-regulated under stress conditions.

Put is the precursor of higher PAs: Spd and Spm are synthesised linking the aminopropyl group from S-adenosylmethionine (SAM) converted to decarboxylated SAM (dcSAM) by SAM decarboxylase, and transferred by spermidine- and spermine- synthase (SPDS e SPMS) (Smith et al. 1979; Borrell et al. 1995). Polyamines also interact with chloroplast metabolic pathways. As an example, because Put can donate amino groups to α-ketoglutarate, PAs may contribute indirectly to glutamate synthesis, a precursor of chlorophyll. Exogenous PAs accelerate the conversion of protochlorophyllide to chlorophyllide and enhance photosynthetic efficiency (Beigbeder et al. 1995). Studies in cucumber etioplasts showed that PA levels on membranes change dynamically during greening, with Put and Spd increasing early and Spm rising later; kinetin further enhances these patterns (Sobieszczuk-Nowicka et al. 2007a, b). Cytokinin-induced chloroplast differentiation may also involve PAs bound to thylakoid membranes (Legocka and Żarnowska 1999). Correlations between PA levels, chlorophyll biosynthesis, and photosynthetic rate have been documented (Beigbeder et al. 1995). PAs stabilise thylakoids (Besford et al. 1993; Legocka and Zajchert 1999), prevent plastid protein degradation under osmotic stress, and in thylakoids are associated with PSII, particularly with light-harvesting complex II (Kotzabasis et al. 1993). Putrescine stimulates ATP synthesis and modulates the proton motive force (pmf), influencing both ΔΨ and ΔpH components (Ioannidis et al. 2012a). Chloroplasts display the earliest visible senescence symptoms due to the degradation of chlorophyll and other pigments (Van Doorn and Yoshimoto 2010). Exogenous PAs delay senescence by reducing chloroplast protein and chlorophyll degradation (Serafini-Fracassini et al. 2010). Polyamines strongly protect photosystems also from stress in a charge-dependent manner and restore photochemical efficiency. Overall, PAs exert multiple effects on plastids, ranging from structural stabilisation to modulation of electron transport and developmental transitions (Loudya et al. 2024). However, the underlying molecular mechanisms are not fully understood.

## Transglutaminases

A major advance in understanding PA function came with the discovery that transglutaminases (TGases), a family of Ca^2^⁺-dependent enzymes, are active in plastids. By catalyzing the covalent attachment of PAs to glutamine residues on target proteins, TGases provide a mechanistic framework for PA involvement in the assembly and regulation of photosynthetic complexes and, ultimately, in sustaining chloroplast bioenergetics. Beyond mediating PA conjugation, TGases participate in protein crosslinking, membrane remodeling, fertilization process and stress- and senescence-related processes (Del Duca et al. 2014; Mandrone et al 2019; Parrotta et al. 2022). Given (i) the biochemical versatility of PAs, (ii) the presence of TGase activity in plastid-containing tissues, and (iii) the high sensitivity of photosynthetic machinery to structural and redox perturbations, a focused synthesis on PA–TGase interactions in chloroplasts is timely.

Transglutaminases (TGases; EC 2.3.2.13) are Ca^2^⁺-dependent acyltransferases, ubiquitously distributed across plant and animal kingdoms, that catalyze the formation of covalent ε-(γ-glutamyl) lysine isopeptide bonds and glutamyl-polyamine conjugates (Fig. 2 a) (Serafini-Fracassini and Del Duca 2008). Besides forming these bonds, that exhibit high resistance to proteolytic degradation, transglutaminases also form extensively cross-linked, generally insoluble, protein biopolymers that are indispensable for the organism to create barriers and stable structures (Martins et al. 2014). Plant transglutaminases share a conserved Cys-His-Asp catalytic triad and exhibit 3D conformational similarity to animal TGases, despite low sequence identity (Beninati et al. 2013; Della Mea et al. 2004a; Parrotta et al. 2022). Evolutionarily, gene duplication in monocots and eudicots led to diversification of TGase function, possibly contributing to unique plant adaptive strategies (Panchy et al. 2016; Parrotta et al. 2022). Bioinformatic analysis of *Arabidopsis thaliana* AtPng1p, the first plant TGase sequenced, revealed expected catalytic and regulatory motifs for Ca^2^⁺ and GTP binding (Della Mea et al. 2004a). AtPng1 knockout mutant of *Arabidopsis thaliana* shows a juvenile phenotype characterized by fewer, smaller and less differentiated cells (Serafini-Fracassini et al. 2021). In maize and rice, chloroplast-localized TGases display strict Ca^2^⁺-dependency and may be regulated by GTP, paralleling animal counterparts but also demonstrating plant-specific features (Campos et al. 2013; Carvajal-Vallejos et al. 2007). TGases have been localized in various cellular compartments; in chloroplasts, they contribute to the remodeling and stabilization of major photosynthetic protein complexes and coordinate responses to physiological stresses (Falcone et al. 1993; Del Duca and Serafini-Fracassini 2005). TGases are present in several plant organelles, and their diversity reflects compartment-specific regulatory functions (Serafini-Fracassini et al. 2009; Martins et al. 2014). TGases have been isolated from both stroma and thylakoid fractions of chloroplasts (Fig. 2b) (Falcone et al. 1993; Ioannidis et al. 2012b). Plastidial TGase isoforms, including the 39 kDa thylakoid-localized variant, display light-dependent activity and catalyze polyamine conjugation to both stromal protein Rubisco and thylakoid membrane proteins (Del Duca et al. 1994; Ioannidis et al. 2012b; Martins et al. 2014). Compartmentalized protein substrates, such as those in thylakoid membranes, suggest specialized regulatory functions. Immunological and biochemical studies in *Helianthus tuberosus* have identified major TGase substrates in isolated and sub-fractionated chloroplasts, further underscoring compartmental control (Dondini et al 2003; Martins et al. 2014). A striking feature is the dependence of chloroplast TGase on light exposure, linked directly to the activation and assembly of photosynthetic supercomplexes (Aloisi et al. 2016; Zhong et al. 2019). Additionally, TGase function itself is pH-dependent, and the plastidial environment’s pH fluctuations, modulated by light and photosynthetic activity, influence the formation and proportions of mono- and bis-γ-glutamyl-polyamine conjugates (Falcone et al. 1993; Martins et al. 2014). TGase-mediated structural modification of chloroplast proteins significantly affects photosynthetic efficiency, plant growth, and delay of senescence (Fig. 2b). Covalent conjugation of spermine to LHCII, catalyzed by thylakoid TGase is light-dependent (Della Mea et al 2004b), and promotes photoprotection by stabilizing photosynthetic complexes (Ioannidis et al. 2016, 2012b; Martins et al. 2014). Over-expression of plastidial TGase leads to pronounced changes in thylakoid architecture, greater grana stacking, expansion of the PSII antenna, and improved resistance to photoinhibition and environmental stress (Ioannidis et al. 2016; Ortigosa et al. 2010). These modifications delay chloroplast dismantling during developmental cell death, likely attributable to spermine-LHCII cross-linking, which protects photosystems and chlorophyll content during senescence (Ioannidis et al. 2012b; Martins et al. 2014). TGase action upon RuBisCo and LHCII not only stabilizes these complexes against degradation but also modulates proton motive force (pmf) partitioning and non-photochemical quenching (qE), fundamentally shaping the energetic and protective responses of the photosynthetic apparatus (Ioannidis et al. 2016, 2012a; Campos et al. 2013).

Polyamines serve as primary physiological substrates for TGase transamidation in chloroplasts (Signorini et al. 1991; Aloisi et al. 2016). Their incorporation into thylakoid proteins not only enhances protein stability, renders them more resistant to proteolysis, and optimizes supramolecular organization, but PAs themselves are effective radical scavengers, bolstering resistance to oxidative stressors such as UV-B and ozone (Martins et al. 2014; Ioannidis et al. 2016). In vitro and in vivo studies confirm that TGase activity, modulated by pH and redox changes, increases with stress or during leaf senescence (Fig. 2b), guiding sequential assembly and controlled degradation of photosynthetic protein complexes (Sobieszczuk-Nowicka et al. 2015; Zhong et al. 2019). Proteomic profiling reveals glutamyl-polyamine derivatives and cross-linked biopolymers in isolated chloroplasts, especially under metabolic or environmental stimuli (Signorini et al. 1991; Del Duca et al 1995). Polyamine-TGase interactions thus support both immediate protective actions and long-term preservation of photosynthetic capacity under stress, linking redox, energy, and metabolic signaling at the suborganellar level (Ioannidis et al. 2012a; Ortigosa et al. 2010; Martins et al. 2014). Activity of chloroplast TGase rises in response to abiotic (e.g., light, salt, temperature, heavy metals) and biotic (pathogen-induced) stressors, strengthening the bioenergetic and structural components of photosynthetic membranes (Jahan et al. 2021; Ortigosa et al. 2010). This contributes to enhanced tolerance, stress signaling, and the regulated onset of programmed cell death (Del Duca et al. 2014).

## Conclusions

Chloroplasts remain at the core of the planetary energy economy, converting solar radiation into biochemical energy and sustaining both the biosphere and human civilisation. Therefore, understanding the molecular factors that preserve the structure and function of chloroplasts is central not only to plant biology, but also to global food security and energy production. Among these regulators, PAs and TGases have emerged as key modulators of photosynthetic efficiency, plastid development, senescence delay and stress resilience. The evidence summarised in this review highlights how PAs contribute through multiple, partially overlapping mechanisms, including thylakoid stabilisation, modulation of proton motive force, regulation of chlorophyll biosynthesis, and protection against oxidative stress, while TGases provide the enzymatic basis for covalent PA-protein conjugation within chloroplast. Their combined actions contribute to maintaining photosynthetic performance under adverse environmental conditions, delaying senescence, and supporting biomass accumulation.

Future research directions should focus on resolving several key knowledge gaps. First, the dynamics and structural consequences of TGase-mediated cross-linking within thylakoid membranes remain not fully understood, particularly in relation to rapid adjustments in photosystem composition and thylakoid membrane remodelling. A second open question concerns the auxiliary catalytic activities of plant TGases, including their possible deamidase functions: although less characterised, such activities could modulate protein charge and conformation, potentially influencing thylakoid architecture and stress-induced signalling. Clarifying these aspects will be essential for understanding how TGases contribute to chloroplast homeostasis under fluctuating environmental conditions. Given their pivotal role at the interface of protein chemistry, redox regulation and stress response, plastidial TGases represent promising targets for genetic and biotechnological strategies aimed at improving crop performance (Parrotta et al. 2022; Zhong et al. 2019). Manipulating TGase activity, alone or in combination with controlled modulation of PA metabolism, could enhance photosynthetic efficiency, strengthen chloroplast structures under heat, drought or salinity, and support yield stability especially in marginal environments. In plants, TGase involvement in hypersensitive responses and disease resistance (Del Duca et al 2007; Del Duca and Serafini-Fracassini 2008) further suggests potential applications opening additional avenues for breeding or engineering crops with improved resilience to pathogens, thereby reducing losses and contributing to food and, more broadly, energy availability.

In conclusion, integrating PAs and TGase research with chloroplast physiology may unlock new strategies to enhance plant productivity and sustainability. As the world faces increasing pressure to secure both sufficient food and clean energy, a deeper understanding of how these molecular systems shape photosynthetic performance will be essential. Advancing chloroplast biochemistry thus remains a pivotal scientific avenue for addressing the intertwined challenges of nutrition, agriculture and global energy.

## Data Availability

No datasets were generated or analysed during the current study.
